# Clinical assessment of dedicated SRS detector for dose verification in SBRT/SRS treatment plans: to establish clinical threshold with AAPM TG-218 recommendation

**DOI:** 10.1007/s11845-026-04379-y

**Published:** 2026-04-14

**Authors:** Cemile Ceylan, Osman Artunç Türe

**Affiliations:** 1 Deparment of Radiation Oncology, Medical Park Istanbul Oncology Hospital, Istanbul, Türkiye; 2 Department of Medical Physics, Faculty of Health Sciences, Yeditepe University, Istanbul, Türkiye; 3 Department of Health Physics, Graduate School of Health Sciences, Istanbul Medipol University, Istanbul, Türkiye

**Keywords:** SRS, SBRT, patient-specific QA, MOS 2D diode array

## Abstract

**Background:**

Stereotactic radiosurgery (SRS) and stereotactic body radiotherapy (SBRT) deliver high radiation doses with steep dose gradients near critical organs, demanding patient-specific quality assurance (PSQA) systems with sub-millimetre spatial resolution beyond conventional criteria.

**Aims:**

This study evaluated a high-resolution complementary metal oxide semiconductor detector for PSQA in SRS and SBRT, focusing on sensitivity to plan modulation and dose gradient accuracy.

**Methods:**

Forty clinical plans (20 SRS, 20 SBRT) were retrospectively analysed and measured using a dedicated stereotactic phantom which is myQA SRS. Gamma analysis was performed with strict spatial criteria. Correlations between gamma passing rates and modulation factor were assessed using Spearman’s rank correlation. Dose gradient accuracy was evaluated by comparing measured and calculated dose profiles.

**Results:**

Mean gamma passing rates for all plans were 95.4% ± 5.1% for 3%/2 mm, 93.6% ± 5.9% for 4%/1 mm, and 89.9% ± 7.4% for 3%/1 mm criteria. A statistically significant inverse correlation between modulation factor and gamma passing rate was observed for stereotactic radiosurgery plans, strongest for the 3%/1 mm criterion (ρ = −0.620, p = 0.0035), while no significant correlation was found for stereotactic body radiotherapy plans. Dose profile analysis demonstrated excellent agreement in steep gradient regions, with coefficients of determination ranging from 0.91 to 0.998.

**Conclusions:**

The high-resolution detector accurately resolves steep dose gradients and small-field penumbras characteristic of stereotactic treatments. The findings support the use of stricter, site-specific QA action limits to fully exploit high-resolution detector capabilities and improve confidence in stereotactic PSQA.

**Supplementary Information:**

The online version contains supplementary material available at https://doi.org/10.1007/s11845-026-04379-y.

## Introduction

Stereotactic Radiosurgery (SRS), Stereotactic Radiation Therapy (SRT), and Stereotactic Body Radiation Therapy (SBRT) are advanced external beam techniques employed to deliver high doses of radiation in a limited number of fractions, thus achieving a high biologically effective dose to targeted intracranial or extracranial tumors [1, 2]. The implementation of robust quality assurance protocols is crucial in SBRT and SRS to ensure the accurate delivery of the prescribed dose and to minimize the risk of complications. When SBRT addresses the complexities arising from the physiological motion of tumours, such as in lung SBRT, and the adjacency of healthy tissues, as seen in spine SBRT cases, SRS applications, particularly when designed for multiple brain metastases, require stringent dosimetric precision for accurate treatment delivery [3, 4]. For vertebral SBRT planning, the area around the spinal cord, which is usually the dose-limiting structure, needs to have a very conformal and sharp dose fall-off. Achieving these clinical objectives often requires substantial modulation during treatment planning to deliver high target doses while respecting constraints for adjacent critical structures such as the spinal cord and normal brain tissue. However, increased plan modulation may introduce greater uncertainty in dose delivery and dosimetric verification for linac-based stereotactic treatments [5]. Patient-specific quality assurance (PSQA), as it evaluates whether the delivered dose accurately matches the planned dose distribution at the treatment planning system (TPS) using a gamma-index pass criteria, is a crucial step of the comprehensive quality control of radiation treatments in general [6]. Establishing clinically meaningful thresholds for pre-treatment verification is therefore essential, particularly in stereotactic applications where geometric and dosimetric precision play a critical role. Recommendations on tolerance limits and QA methods are provided in the TG218 report [7], which included a universal action limit of 3%/2 mm with a 10% threshold and a 90% passing rate for standard Intensity Modulated Radiation Therapy (IMRT) and Volumetric Modulated Arc Therapy (VMAT), while tighter tolerances may be warranted without giving any specifics for SRS and SBRT cases. A tolerance limit of 3% for dose criteria and a 1 mm distance to agreement, associated with the performance characteristics of PSQA equipment, should be adopted for SRS-SBRT in clinical practice. This approach is essential for radiosurgery, which necessitates increased doses, reduced margins, and steep dose gradients, achievable in patient treatment delivery with high precision. Due to the small dimensions and steep high-dose gradients of photon beams used for SRS/SBRT treatments, TG 101 recommends using dosimeters with a spatial resolution of approximately 1 mm or better to measure basic dosimetry data along with detailed dose correction factors applied [8]. Consequently, a high-resolution detector is recommended for PSQA of SRS and SBRT plans to verify that the dose gradient between the target and the adjacent critical organs can be delivered as planned [9]. Numerous studies have suggested that tighter gamma criteria such as 3%/2 mm, 3%/1 mm, and 4%/1 mm-are suitable for PSQA of SRS and SBRT plans when measurements are performed using detectors specifically designed for radiosurgery, including radiochromic film or dedicated high-resolution array systems such as the myQA SRS (IBA Dosimetry, Germany) or the SRS MapCHECK (Sun Nuclear Corporation, Melbourne, FL, USA) [10–12]. These studies consistently highlight the influence of detector size and spatial resolution on gamma analysis outcomes, particularly in high-gradient regions. Although radiochromic film is routinely employed for PSQA because of its excellent spatial resolution and its ability to capture dose distributions in arbitrary anatomical planes, its clinical use is often constrained by practical limitations. Film dosimetry requires meticulous handling, controlled scanning conditions, batch-specific calibration, and extended processing time; furthermore, it is sensitive to scanner non-uniformity, orientation effects, and potential user-dependent variability [13]. These limitations reduce workflow efficiency and make film less ideal for high-throughput clinical environments, particularly when frequent verifications are required. In response to these limitations, new high-resolution detectors specifically designed for radiosurgery PSQA have been developed and found their place in the clinic workflow of PSQA. Such systems provide improved spatial sampling, reduced volume-averaging effects, and rapid readout, making them more suitable for routine SRS and SBRT pre-treatment dose verification, as well as for point dose measurements and comparisons with film measurements using separate ion chamber holders that are appropriate for small field dosimetry and EBT3 film [14, 15]. In a recent study, we reported initial clinical experience with a submillimeter-resolution complementary metal oxide semiconductor (CMOS) detector for radiosurgery QA. The present study retrospectively analyzes a cohort of SRS and SBRT cases measured using the myQA SRS detector and SRS phantom in our clinic. The aims of this study are to establish clinically achievable gamma passing rates under TG-218 and TG-101 guidance, to investigate the relationship between gamma analysis results and plan complexity metrics such as modulation factor, and to assess detector performance in steep dose-gradient regions relevant to stereotactic treatments.

## Materials and methods

### CT scan of IBA SRSPhan detector and electron density override

The IBA SRSPhan contains a high-resolution CMOS detector array embedded within an ABS (Acrylonitrile-Butadiene-Styrene) phantom housing. ABS is a mechanically stable, tissue-equivalent thermoplastic with a relative electron density of approximately 1.048, making it suitable for CT imaging and Monte Carlo-based dose calculation without introducing high-density artifacts. The CMOS architecture enables very small pixel structures, low electronic noise, and rapid readout, yielding a 0.4 mm spatial resolution across a 12 × 14 cm² active area. The absence of inter-detector spacing allows direct sampling of the dose distribution, thereby eliminating the need for spatial interpolation and improving measurement accuracy. This sub-millimetric resolution matches the approximately 1-mm or superior spatial accuracy recommended by AAPM TG-101 for stereotactic dosimetry and overcomes the detector performance assumptions underlying the tighter gamma criteria stated in AAPM TG-218 for PSQA of SRS/SBRT.

**Fig. 1:**
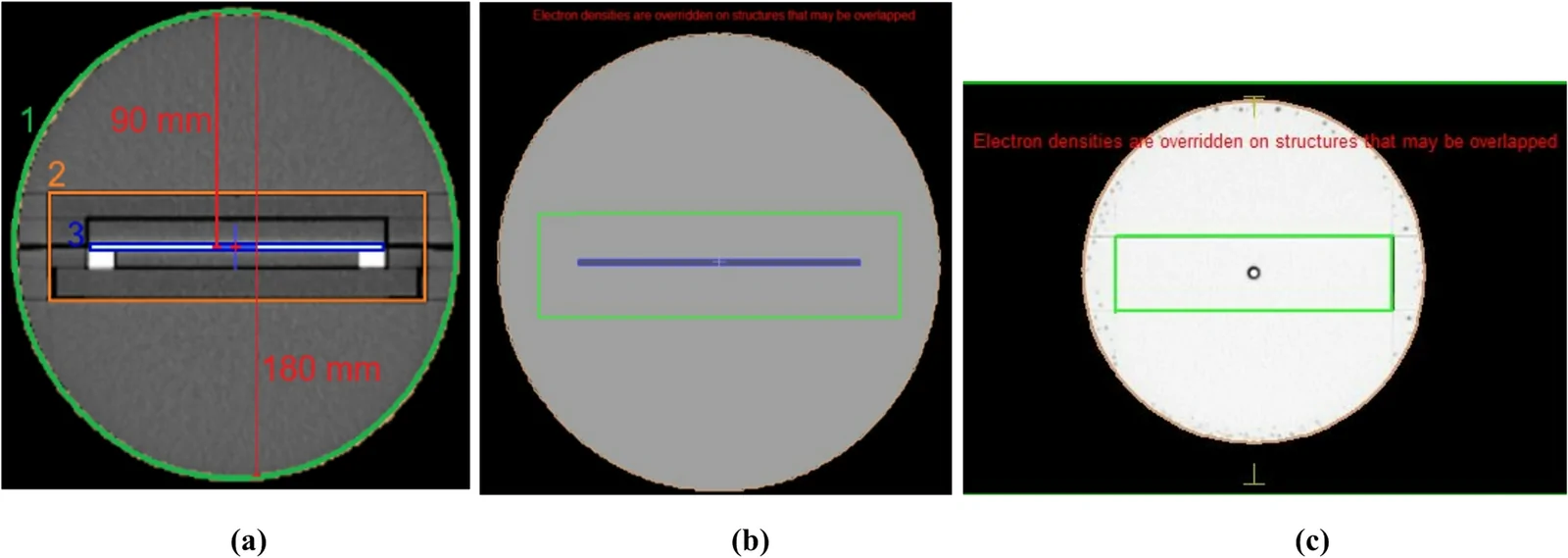
CT axial section of SRSPhan. **a** Dimension of delineated ABS and detector (**b**) The synthetic CT images of the SRSPhan with overriding Relative Electron Density (RED), (**c**) Ion chamber location into the inserted plug of SRSPhan

The SRSPhan was scanned using a 1.25 mm CT slice thickness, and all relevant components-including the ABS housing, CMOS detector layer, and insert compartments-were contoured in the TPS to verify geometric consistency. The central detector pixel was aligned to the treatment isocenter using the planning CT. Figure 1a represents the dimensions of the detector featuring the outlined ABS and detector on the TPS, whereas Fig. 1b presents the axial planar image of the synthetic CT images with the overriding suggested relative electron density of the ABS and detector.

**Fig. 2:**
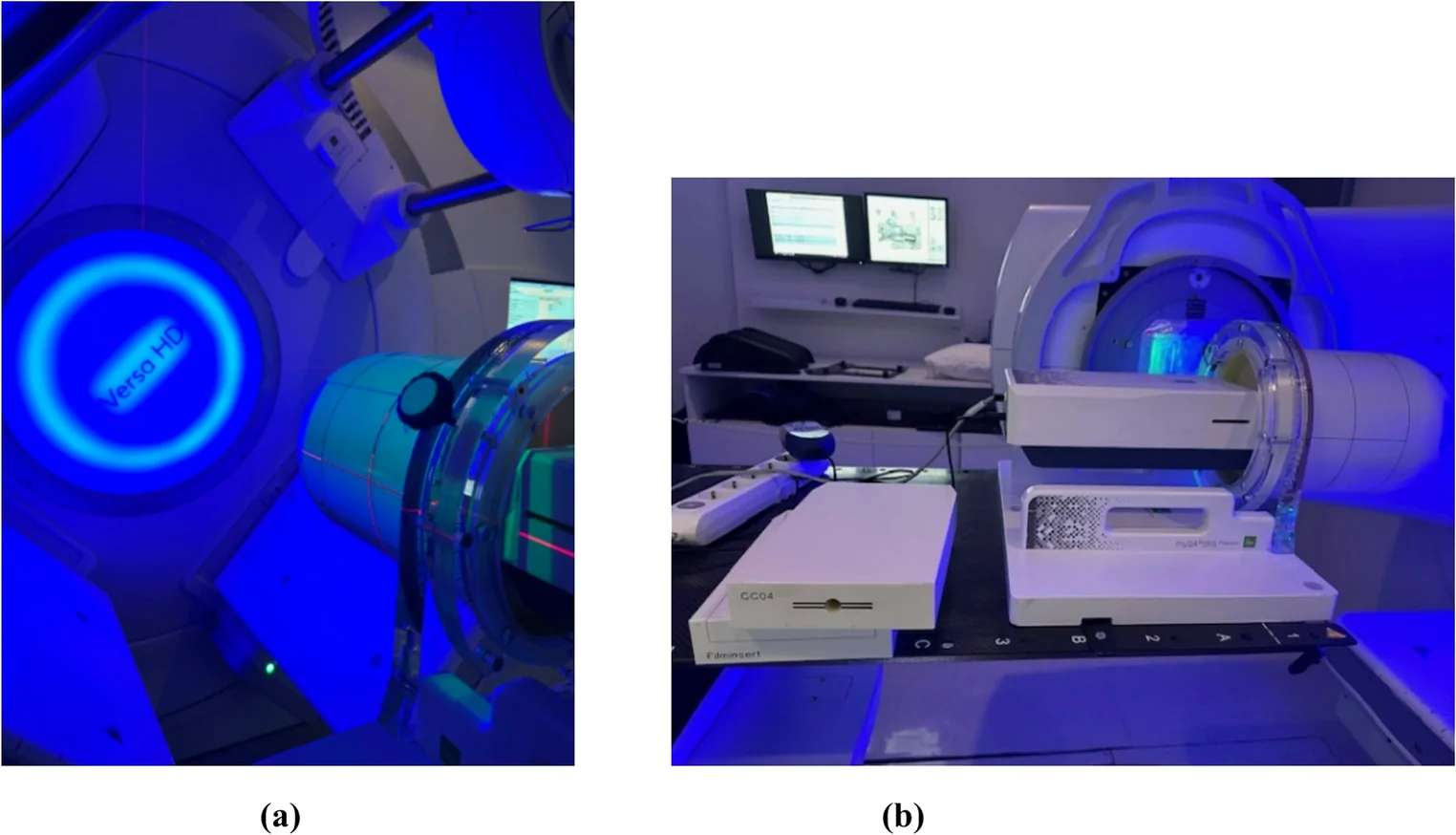
The alignment of the SRSPhan center to isocenter of the Linac (**a**) and (**b**) embedded CMOS detector and chamber plug for the CC04 ion chamber

Electron-density overrides were applied for Monte Carlo dose calculation, following IBA recommendations [15], because the detector’s internal components differ from human tissue and include electronic layers that automated CT-to-density conversion does not adequately represent. The CMOS sensor region was overridden to 1.00 g/cm³, corresponding to 0 Hounsfield Number (HU), and the ABS phantom body to 1.048 g/cm³, corresponding to 45 HU, ensuring accurate modelling of attenuation and scatter as shown in Fig. 1. These overrides prevent dose-calculation biases that may otherwise arise from non-water-equivalent electronic structures. Before measurement, CBCT-guided alignment was performed to register the phantom with the planning CT and correct residual setup deviations, ensuring consistency between planned and delivered geometry during all SRS/SBRT pre-treatment verifications. Figure 2a and b depict the positioning of the myQA SRSPhan and detector on the linac couch, ensuring that the phantom is aligned on the treatment couch to mitigate the impact of the couch’s attenuation on the detector data.

### Small field consistency and output calibration

Small-field dosimetry was evaluated before performing patient-specific SRS/SBRT verifications to ensure consistency between TPS-calculated output factors and measured values. For reference measurements, a CC04 ion chamber was placed in the dedicated ion-chamber insert of the SRSPhan phantom (Fig. 1c), and absolute dose was measured for 1 × 1, 2 × 2, 3 × 3, 4 × 4, 5 × 5, and 10 × 10 cm² field sizes. The ion chamber setup and corresponding CT slice of the chamber positioning are shown in Fig. 1c. These measurements were compared to TPS-calculated dose values to confirm the accuracy of the small-field beam model used for the SRS/SBRT plans. Following verification of small-field output consistency with the CC04 chamber, the 10 × 10 cm² field was used as the reference field for output calibration of the SRSPhan detector. This step provided the normalization required for subsequent planar dose measurements with the CMOS array to achieve an absolute dose distribution that will be used for direct comparison with the TPS calculation.

### SRS/SBRT plan selection and plan complexity metrics

A total of 40 clinical stereotactic treatment plans were included, consisting of 20 SRS, which consisted of 18 single brain metastases and 2 multiple brain metastases with single isocenter plans, and 20 SBRT cases retrospectively selected from previously treated patients who had already met institutional acceptance criteria. All treatment plans were made with VMAT and 6 FFF energy, using a grid size of 0.1 cm for SRS cases and 0.2 cm for SBRT cases.

Beam geometry varied according to clinical intent and anatomical site. All SRS plans were delivered with non-coplanar VMAT arcs, as is standard for intracranial stereotactic treatments requiring steep dose fall-off and highly conformal isodose distributions. In contrast, most SBRT plans were coplanar, with only two SBRT cases using non-coplanar arcs. Nevertheless, several SBRT cases-particularly spine SBRT-featured very high dose gradients adjacent to critical structures such as the spinal cord. These high-gradient spine plans generally raise dosimetric and verification issues comparable to intracranial SRS, despite primarily coplanar beam delivery. The inclusion of both spine and non-spine SBRT cases facilitated a comprehensive assessment of detector performance across various therapeutically pertinent dose gradients and geometries. Key planning data were collected from the TPS for each plan, including prescription dose and fractionation, GTV and PTV volumes, number of VMAT arcs, and beam geometry. The attributes of the SRS (intracranial) and SBRT (extracranial) verification plans, encompassing anatomical locations, average target volumes, beam configuration, and prescription ranges, are encapsulated in Table 1. The mean GTV increased from 1.96 cc in SRS to 15.31 cc in SBRT, with corresponding mean PTV of 3.90 cc and 30.80 cc, reflecting the substantially larger target volumes in extracranial stereotactic treatments.

**Table 1:** Summary of patient cohorts and treatment characteristics for stereotactic radiosurgery (SRS) and stereotactic body radiotherapy (SBRT) plans included in this study. The table reports the number of plans, treated anatomical sites, mean gross tumor volume (GTV) and planning target volume (PTV), beam geometry (co-planar versus non-coplanar), prescribed dose ranges, and fractionation schemes for each treatment group

Group	Number of plans	Treatment sites	GTV (cc) (mean ± SD, range)	PTV (cc) (mean ± SD, range)	Beam geometry (Co-planar / Non-coplanar)	Prescription dose (Gy)	Fraction (Fx)
SRS	20	Brain metastases	1.96 ± 3.28 (0.12–15.25)	3.90 ± 5.64 (0.55–26.09)	0 / 20 3.9022381	15–25	1–5
SBRT	20	Lung, spine, Adrenal Glands, Lymph node, Bone	15.31 ± 11.42 (1.01–39.54)	30.80 ± 16.40 (6.72–62.70)	18 / 2	18–60	1–8

The plan complexity of each treatment plan was quantified using the Modulation Factor (MF). According to the methodology established by Hernandez et al. [16], plan complexity is a pivotal factor influencing “plan quality,” indicating the extent of beam aperture modulation necessary for attaining conformal dose distributions. In this study, MF is defined as the ratio of the total monitor units (∑MU) necessary to administer the plan to the specified dose per fraction (Dpresc):

$$\mathrm{MF}=\:\frac{\sum\:\mathrm{M}\mathrm{U}}{Dpresc}$$

In this formulation, the prescription dose serves as the normalization reference, allowing for a standardized comparison of modulation levels across plans with different prescribed dose levels. The MF values were obtained directly from the Monaco treatment planning system (TPS), which provides this numerical data in real-time during plan optimization and finalizes it after the segmentation process. MU-normalized metrics such as MF are widely recognized as practical surrogates for delivery complexity, as highly modulated VMAT) plans generally require smaller, more irregular apertures and more complex multileaf collimator (MLC) motion. Previous research has demonstrated that increasing plan complexity can reduce gamma passing rates and enhance sensitivity to delivery and modeling uncertainties [17]. To systematically investigate this relationship, plans were categorized into descriptive ranges of low (MF < 3.0), intermediate (3.0 ≤ MF < 5.0), and high (MF ≥ 5.0) modulation. These thresholds were established based on the observed distribution within this cohort to identify plans with excessive modulation, consistent with international guidelines and the AAPM TG-218 recommendation to document modulation ranges to prevent verification failures. Additionally, the Gradient Index (GI) was utilized to characterize the dose fall-off outside the target volume, which is particularly critical in regions of high dose gradient. Following the standard definition by Paddick [18], GI is expressed as the ratio of the 50% prescription isodose volume (V50%) to the prescribed isodose volume (Vpresc):

$$\mathrm{GI}=\frac{V50{\%}}{Vpresc}$$

By evaluating both MF and GI, this study enabled a systematic assessment of how plan complexity and dose gradient characteristics influence dosimetric verification performance under TG-218 and TG-101 evaluation criteria.

### Measurements with the myQA SRS detector, gamma, and dose gradient analysis

All selected SRS and SBRT treatment plans were recalculated on the CT dataset of the myQA SRSPhan. The original patient plans were rigidly mapped onto the phantom geometry, and the isocenter was positioned at the geometric center of the delineated CMOS detector layer, ensuring that the region of highest detector resolution coincided with clinically relevant high-dose and high-gradient regions. Before irradiation, the myQA SRSPhan was placed on the treatment couch by using the dedicated indexing system and aligned with the planning CT through image-guided radiotherapy (IGRT). Translational and rotational setup errors were corrected before delivery. Same-day linac output correction was applied prior to each measurement session to account for daily output variations. All measurements were performed using 6 FFF photon beams, consistent with the clinical treatment plans. During delivery, the integrated gantry-angle sensor of the myQA SRS system was enabled to record beam incidence angles. Angular dependence correction was implemented with manufacturer-provided look-up tables (LUTs) aligned with the measured TPR value of the 6 FFF beam, hence enabling precise dose reconstruction for both coplanar and non-coplanar VMAT delivery.

Measured dose distributions were compared with TPS-calculated dose distributions in Monaco using the same dose grid resolution as the original clinical plans to avoid additional spatial interpolation uncertainty. Treatment plans were calculated using a dose calculation grid of 1 mm for SRS plans and 2 mm for SBRT plans, reflecting the higher spatial resolution required for intracranial stereotactic radiosurgery compared with extracranial stereotactic body radiotherapy. Plan agreement was evaluated using the gamma passing rate (GPR), defined as the percentage of evaluated detector points satisfying the selected gamma criteria (γ < 1). Gamma analysis was performed using the myQA Patients software (IBA Dosimetry, Germany) through a volume-based comparison using the TPS-calculated RTDOSE distribution as the reference dose volume. The measured detector dataset was automatically registered to the TPS reference dataset prior to evaluation. To ensure spatial correspondence between the TPS dose grid and detector sampling points, bilinear interpolation of the calculated dose grid was applied during gamma computation, as implemented in the myQA Patients analysis algorithm.

Gamma evaluation was performed in global absolute-dose mode, with the TPS dose distribution used for normalization and a 10% low-dose threshold, consistent with the recommendations of AAPM TG-218 for measurement-based patient-specific quality assurance. Gamma criteria of 3%/1 mm and 4%/1 mm were applied together with 3%/2 mm to assess sensitivity to both dosimetric and spatial tolerances. The stricter 1 mm distance-to-agreement criterion was used to increase sensitivity to spatial discrepancies in steep dose-gradient regions typical of SRS and high-gradient SBRT treatments, whereas the 3%/2 mm criterion was included to evaluate overall robustness under moderately relaxed spatial conditions. A gamma search distance of 4.5 mm was used, exceeding the largest distance-to-agreement criterion applied in the analysis and ensuring that the algorithm could identify the optimal comparison point within the search neighbourhood without truncation of the gamma calculation. This combined evaluation strategy ensured that variations in GPR primarily reflected true dosimetric and spatial agreement rather than artifacts arising from overly permissive gamma tolerances.

The use of fine TPS voxel sizes together with interpolation is particularly important for stereotactic verification, as coarse dose grids may introduce spatial discretization effects that influence gamma evaluation in steep dose-gradient regions. Previous studies evaluating detector-array–based quality assurance in stereotactic radiotherapy have demonstrated that improved spatial resolution in both dose calculation and measurement domains enhances the sensitivity of gamma analysis when strict spatial criteria such as 3%/1 mm or 2%/1 mm are applied.

In addition to array-based measurements, radiochromic film dosimetry was performed using EBT3 film (Ashland Specialty Ingredients) to investigate potential discrepancies between measurements obtained with the myQA SRS detector and those acquired using a film insert plug within the SRSPhan (Fig. 2b). This comparison was limited to three representative SRS cases, including two single-isocenter multiple-brain-metastases plans and one single-lesion SRS plan. The single-lesion case exhibited a relatively high modulation factor and a small GTV (MF = 5.82, GTV = 0.37 cc), making it particularly sensitive to spatial resolution and high-gradient effects.

To further assess detector performance in steep dose fall-off regions, dose profile comparisons were performed for five SRS and five SBRT plans. One-dimensional dose profiles were extracted from both TPS-calculated and myQA SRS-measured dose distributions across clinically relevant high-gradient regions, such as PTV edges and PTV-organ-at-risk interfaces (e.g., spinal cord in spine SBRT). The consistency between calculated and measured dose profile values was assessed by linear regression analysis, calculated as

$$\:Dmeas=a\hspace{0.17em}DTPS+b$$

where DTPS and Dmeas represent the TPS-calculated and measured dose values, respectively. The coefficient a denotes the slope and indicates the degree of concordance between measured and TPS-calculated dose values, with values near unity signifying precise dose reproduction by the myQA SRS detector. The parameter b signifies the intercept and indicates a systematic dose offset, with values near zero reflecting negligible bias between measured and estimated doses. The coefficient of determination (R^2^) was used to quantify agreement, with values approaching unity indicating accurate reproduction of steep dose gradients by the detector that quantify the agreement between measured and TPS-calculated dose profiles in high-gradient regions.

Statistical analyses were performed to examine the relationship between patient-specific QA performance, plan complexity, and dose gradient reproducibility. The relation between the GPR from myQA SRS measurements and plan complexity, quantified by the MF, was evaluated using Spearman’s rank correlation analysis, selected due to the non-normal distribution typically observed in PSQA datasets. The detector’s ability to reproduce steep dose gradients was assessed by comparing measured dose distributions with those computed by the treatment planning system in high-gradient regions, with the degree of concordance established by linear regression analysis. A p-value less than 0.05 was established as the threshold for addressing statistical significance.

## Results

### Findings of small field consistency and output calibration

**Fig. 3:**
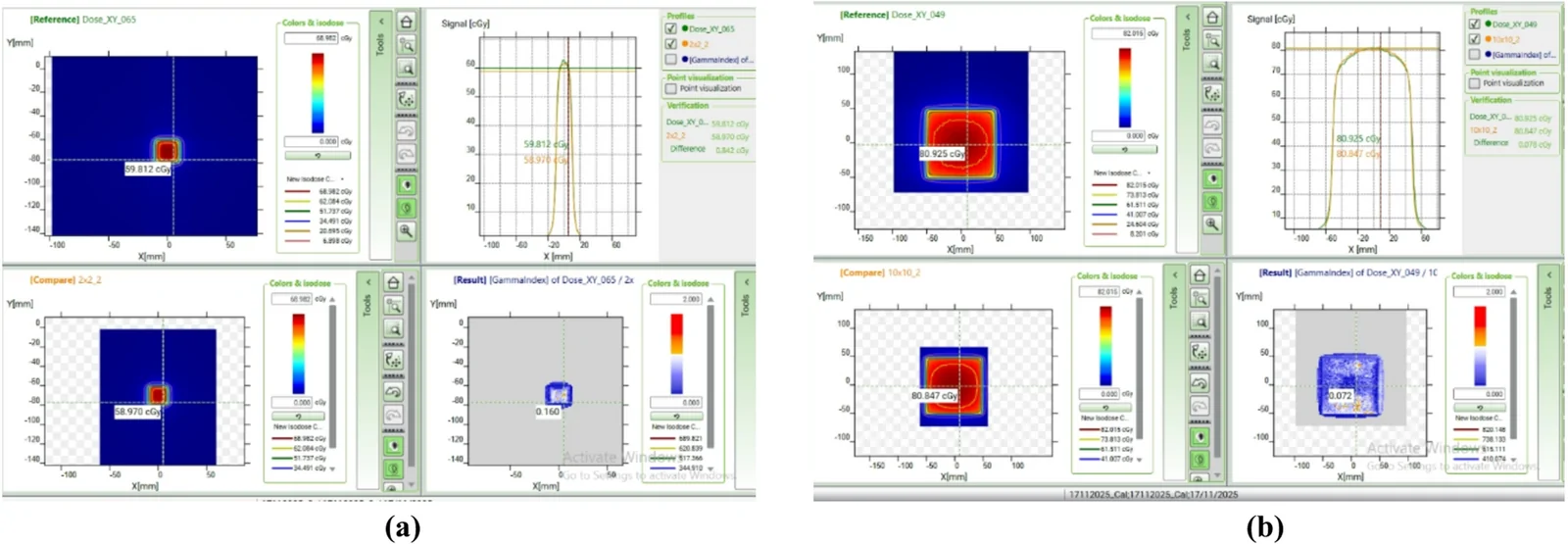
Comparison of TPS-calculated and myQA SRS-measured dose distributions for the (**a**) 2 × 2 cm² and (**b**) 10 × 10 cm² fields. Planar dose maps, dose profiles, and gamma index results are shown. Gamma analysis was performed using the 3%/1 mm criterion with a 10% dose threshold, demonstrating good agreement for both field sizes

Small-field output consistency was assessed prior to patient-specific SRS/SBRT QA to verify agreement between TPS-calculated and measured doses. Absolute point-dose measurements with a CC04 ionization chamber were performed without the application of small-field correction factors, as these measurements were utilized exclusively as a consistency check of the TPS beam model. Absolute point-dose measurements with a CC04 ionization chamber exhibited remarkable concordance for clinically pertinent fields, with average discrepancies of less than 0.8% for the 2 × 2 cm² field and below 0.1% for the 10 × 10 cm² reference field. The results confirmed the accuracy of the TPS beam model and supported the use of the 10 × 10 cm² field for output calibration of the SRSPhan detector. The most significant divergence was noted for the 1 × 1 cm² field (1.4%), aligning with established restrictions in small-field dosimetry. Following output calibration of the detector, planar dose measurements obtained for 2 × 2, 3 × 3, 4 × 4, 5 × 5, and 10 × 10 cm² field sizes with the myQA SRSPhan detector showed significant agreement with dose distributions computed by the TPS. Figure 3 illustrates that the dose profile comparisons for the 2 × 2 cm² and 10 × 10 cm² fields exhibited strong concordance in both the field and penumbra regions. Gamma analysis with the 3%/1 mm criterion produced passing rates of 97.8% for the 2 × 2 cm² field and 99.8% for the 10 × 10 cm² field, demonstrating significant spatial and dosimetric consistency.

### Patient-specific QA results based on gamma passing rate

PSQA results were quantified using the GPR performing in the IBA myQA software for all SRS and SBRT treatment plans measured with the myQA SRS detector. GPR was evaluated using 3%/1 mm, 4%/1 mm, and 3%/2 mm criteria in absolute dose mode with a 10% low-dose threshold, in accordance with AAPM TG-218 recommendations. A summary of the GPR results is provided in Table 2. Both cohorts demonstrated strong overall compliance with the universal TG-218 90% action limit under the 4%/1 mm and 3%/2 mm tolerances; however, agreement consistently decreased as spatial criteria became more stringent.

**Table 2:** Summary of plan complexity, dose gradient, and gamma passing rate results for SRS and SBRT cohorts. Values are reported as mean ± standard deviation (%)

Plan Group	Modulation Factor (MF)	Gradient Index (GI)	GPR 3% / 1 mm (%)	GPR 4% / 1 mm (%)	GPR 3% / 2 mm (%)
SRS (*n* = 20)	4.26 ± 1.05	4.76 ± 1.39	91.1 ± 7.4	94.0 ± 6.9	96.1 ± 4.8
SBRT (*n* = 20)	4.64 ± 1.04	4.10 ± 0.72	88.6 ± 7.4	93.4 ± 5.6	94.7 ± 5.3
All Plans (*n* = 40)	4.45 ± 1.05	4.43 ± 1.15	89.9 ± 7.4	93.6 ± 5.9	95.4 ± 5.1

For the SRS plans (*n* = 20), the mean GPR was 91.1% when using the 3%/1 mm criterion. This increased to 94.0% for the 4%/1 mm criterion and reached 96.1% for the 3%/2 mm criterion. In contrast, the SBRT plans (*n* = 20) showed slightly lower agreement with the strictest criterion. The mean GPR was 88.6% at 3%/1 mm, which improved to 93.4% and 94.7% for the 4%/1 mm and 3%/2 mm criteria, respectively. Across both cohorts, the lowest GPR values and the largest variability were observed under the 3%/1 mm criterion, whereas relaxation of the gamma criteria resulted in consistently higher passing rates and reduced dispersion. When all plans were considered collectively (*n* = 40), the mean GPR increased from 89.9% at 3%/1 mm to 95.4% at 3%/2 mm, demonstrating a clear dependence of passing rates on spatial tolerance.

Regarding plan characteristics, SRS plans demonstrated marginally reduced modulation (MF = 4.26 ± 1.05) alongside elevated gradient index values (GI = 4.76 ± 1.39), which suggests a more pronounced dose fall-off relative to SBRT (MF = 4.64 ± 1.04, GI = 4.10 ± 0.72). These observations imply that although both plan categories satisfied clinically acceptable standards as per TG-218 guidelines, more stringent spatial criteria are more responsive to plan modulation and high-gradient dose areas.

**Fig. 4:**
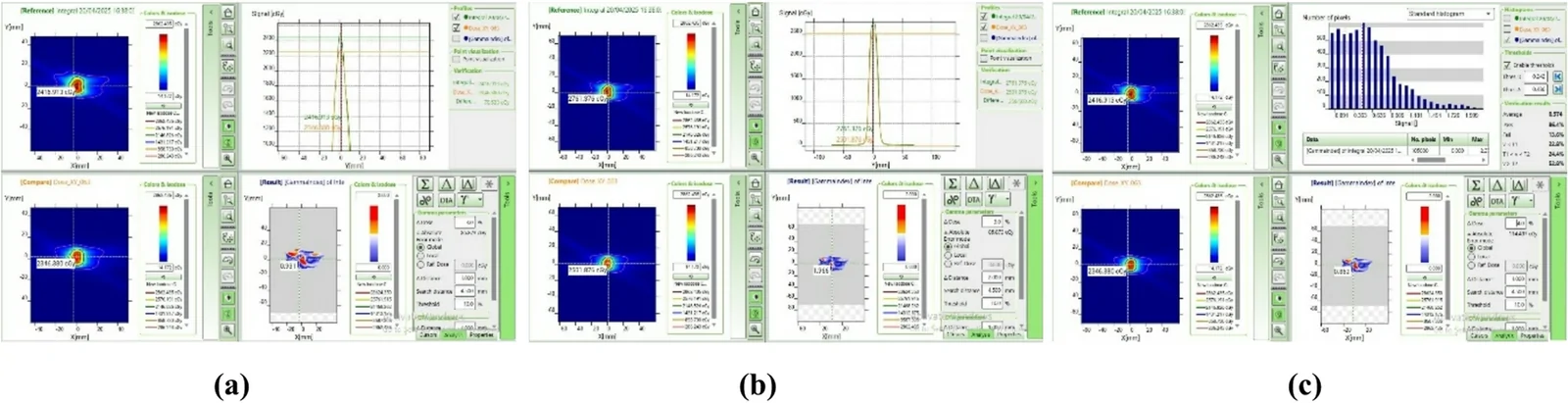
Representative gamma analysis for a representative SRS case (12). Panels (**a**), (**b**), and (**c**) display comparisons between measured and TPS-calculated dose distributions, profiles, and gamma index maps for 3%/1 mm, 3%/2 mm, and 4%/1 mm criteria, respectively

**Fig. 5:**
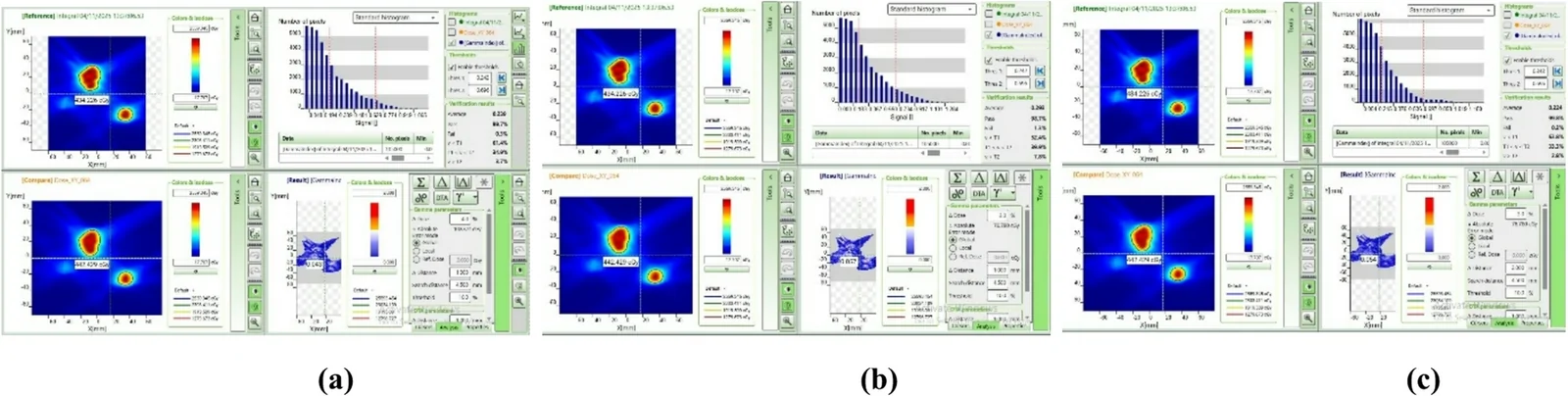
Gamma analysis for a representative SRS case (MF = 4.56) characterized by steep dose gradients. Panels (**a**-**c**) illustrate comparisons between measured and TPS-calculated dose distributions using 3%/1 mm, 4%/1 mm, and 3%/2 mm criteria, respectively. Accompanying gamma index maps and histograms highlight the high concordance in the central high-dose region and localized discrepancies primarily restricted to the target periphery

Evaluation of the measured GPR values relative to the AAPM TG-218 universal action limit of 90%, defined as the percentage of evaluated points with γ < 1, showed that the majority of both SRS and SBRT plans achieved GPR ≥ 90% when evaluated using 3%/2 mm and 4%/1 mm criteria. In contrast, a larger proportion of plans fell below this threshold when evaluated using the more stringent 3%/1 mm criterion. This behaviour is illustrated in Fig. 4 for a plan with relatively high modulation (MF = 5.82), where gamma failures are primarily confined to regions of steep dose gradients, while overall agreement improves with relaxation of either the dose-difference or distance-to-agreement criterion. In contrast to Fig. 4, which illustrates an SRS case with relatively lower gamma passing rates associated with higher modulation and more pronounced spatial discrepancies, Fig. 5 presents an SRS case with a moderate modulation factor (MF = 4.56), small target volume, and high dose gradients, but demonstrating improved overall gamma agreement. Under the 3%/1 mm criterion (Fig. 5a), localized regions with γ ≥ 1 are again primarily confined to steep dose-gradient regions surrounding the target, while the central high-dose region shows close agreement between the TPS-calculated and myQA SRS-measured dose distributions. Compared with Fig. 4, the extent and magnitude of gamma failures are reduced. When the gamma criteria are relaxed to 4%/1 mm and 3%/2 mm (Fig. 5b and c), a further reduction in gamma values is observed, accompanied by an increased fraction of points satisfying γ < 1. The corresponding gamma histograms demonstrate a shift toward lower gamma values, indicating improved agreement across the evaluated plane relative to the case shown in Fig. 4.

Quantitative comparison of GPRs for the three SRS cases with film measurements (SRS7, SRS18, and SRS20) is summarized in Table 3. For SRS18, myQA SRS and film measurements yielded GPRs of 98.7% vs. 97.7%, 99.7% vs. 99.2%, and 99.8% vs. 99.5% for the 3%/1 mm, 4%/1 mm, and 3%/2 mm criteria, respectively. For SRS20, lower overall agreement was observed for both techniques, with myQA SRS GPRs of 79.3%, 86.4%, and 93.9%, compared with 80.5%, 87.4%, and 94.1% obtained using film. Across all three cases, the lowest GPRs were consistently observed under the more stringent 3%/1 mm criterion, with improved agreement under the 3%/2 mm criterion for both measurement techniques.

**Table 3:** Comparison of gamma passing rates (GPR) obtained using the myQA SRS detector and EBT3 radiochromic film dosimetry for three selected SRS cases evaluated under gamma criteria of 3%/1 mm, 4%/1 mm, and 3%/2 mm. Gamma passing rate is defined as the percentage of evaluated points satisfying γ < 1 using absolute dose comparison with a 10% dose threshold

Plan	myQA SRS GPR (3% / 1 mm) [%]	myQA SRS GPR (4% / 1 mm) [%]	myQA SRS GPR (3% / 2 mm) [%]	Film GPR (3% / 1 mm) [%]	Film GPR (4% / 1 mm) [%]	Film GPR (3% / 2 mm) [%]
SRS7	95.0	96.5	98.6	93.2	95.3	97.8
SRS18	98.7	99.7	99.8	97.7	99.2	99.5
SRS20	79.3	86.4	93.9	80.5	87.4	94.1

**Fig. 6:**
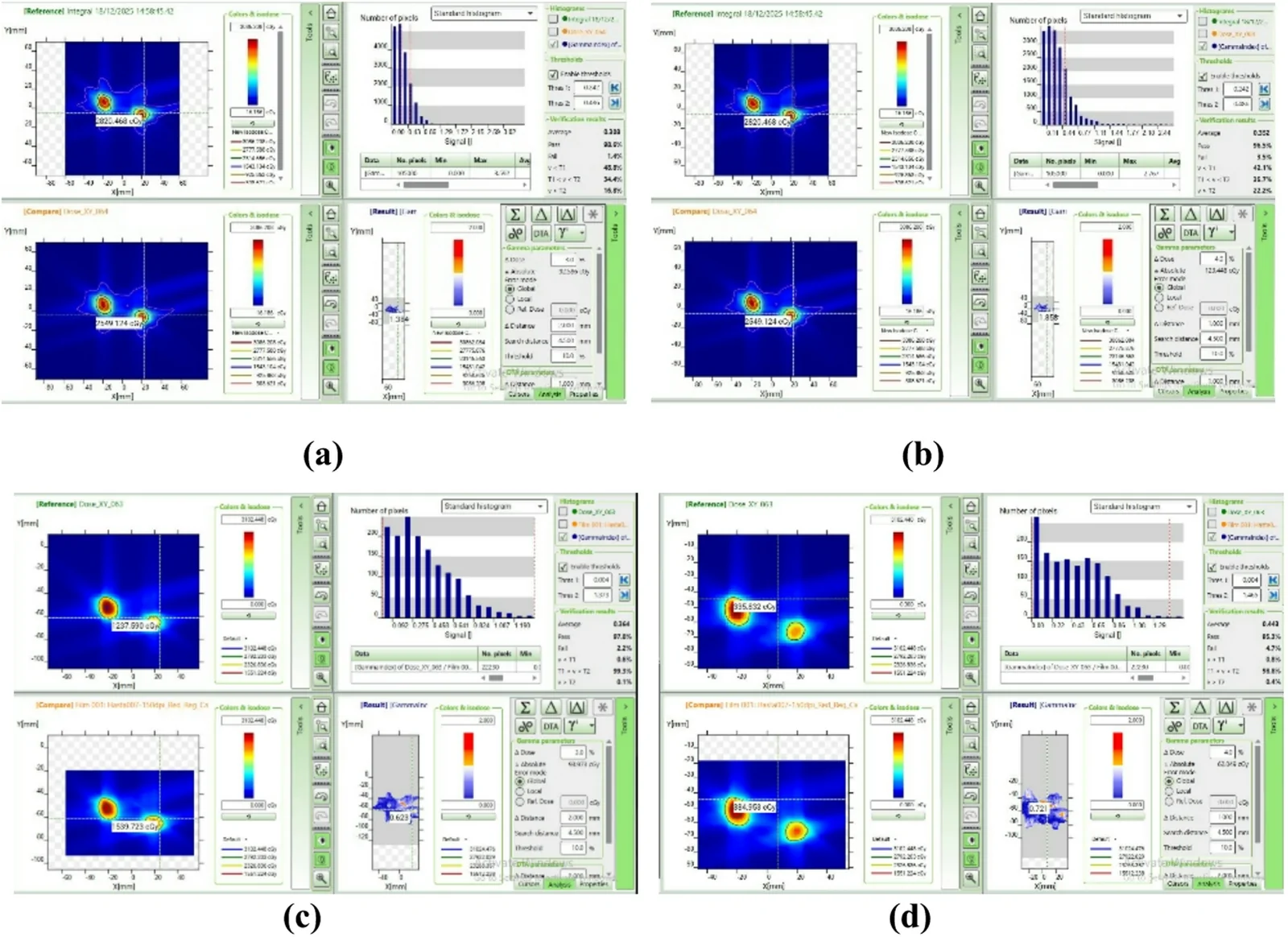
Comparative dosimetric evaluation of the myQA SRS detector and radiochromic film for a highly modulated SRS case (MF = 6.09). Panels (**a**-**b**) show detector-based results and (**c**-**d**) show corresponding film results for 4%/1 mm and 3%/2 mm criteria

As illustrated in Fig. 6, the comparison between EBT3 radiochromic film and myQA SRS detector measurements for SRS7, a multiple-brain-metastases case characterized by a high modulation factor (MF = 6.09) and small target volumes, demonstrates close agreement between the two dosimetry methods. Both film- and myQA SRS-based gamma analyses show highly similar spatial distributions of gamma agreement, with localized gamma failures predominantly confined to steep dose-gradient regions surrounding the targets, while the central high-dose regions exhibit good agreement with the TPS-calculated dose distribution. For SRS7, GPRs obtained with myQA SRS were 95.0%, 96.5%, and 98.6% for the 3%/1 mm, 4%/1 mm, and 3%/2 mm criteria, respectively, compared with 93.2%, 95.3%, and 97.8% from film measurements, corresponding to absolute differences of 1.8%, 1.2%, and 0.8%.

The clinical performance of the high-resolution CMOS array was benchmarked against the institutional gold standard using paired myQA SRS and EBT3 film-based GPR values (*n* = 9; three representative SRS cases evaluated at 3%/1 mm, 4%/1 mm, and 3%/2 mm). Spearman rank correlation analysis yielded a perfect positive monotonic relationship (ρ = 1.00), demonstrating that both dosimetry platforms provide an identical ranking of plan delivery agreement across all evaluated criteria. Consequently, both systems consistently identified plans requiring further investigation, such as SRS20, which exhibited the lowest passing rates on both platforms. Moreover, the significant spatial concordance evident in the compared gamma maps (Fig. 6) confirms that the detector’s sub-millimeter resolution accurately resolves the same localized, gradient-induced discrepancies as radiochromic film. While film dosimetry was restricted to this subset due to its time-consuming processing workflow and resource requirements, this strong cross-modality agreement substantiates the detector’s capability to capture the complex dose fall-offs characteristic of highly modulated stereotactic plans.

**Fig. 7:**
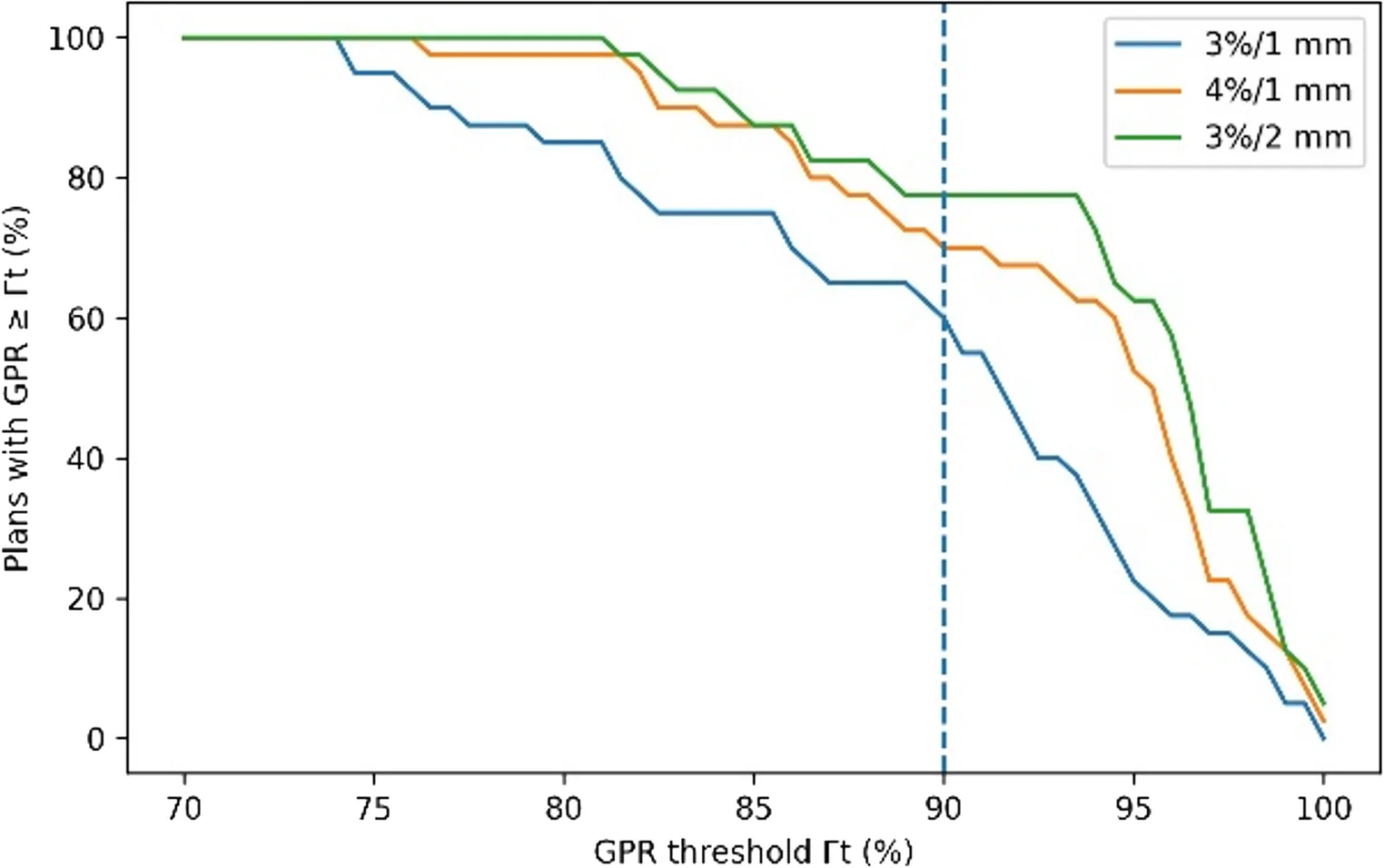
Cumulative gamma passing rate (GPR) compliance for all SRS and SBRT plans evaluated with the myQA SRS detector under the 3%/1 mm, 4%/1 mm, and 3%/2 mm gamma criteria. The curves represent the percentage of plans achieving a GPR greater than or equal to a given threshold (Γt). The vertical dashed line indicates the AAPM TG-218 universal action limit of 90%, defined as the minimum acceptable percentage of evaluated points with γ < 1

Overall compliance with TG-218 action limits across all evaluated plans is illustrated in Fig. 7, which displays cumulative GPR compliance as a function of the passing rate threshold (Γt), with each curve indicating the proportion of plans achieving or exceeding a specified passing level for each evaluated criterion. As shown in Fig. 7, a higher proportion of plans achieved GPR ≥ 90% under the 4%/1 mm and 3%/2 mm criteria compared with the 3%/1 mm criterion, indicating increased robustness of these evaluation settings for both SRS and SBRT plans. These results provide the necessary institutional verification that the CMOS array accurately reproduces the high-gradient distributions required for intracranial SRS while highlighting that the universal 90% threshold may be insufficiently sensitive when using stringent 1 mm spatial tolerances. Evaluation at Γt = 90% directly reflects compliance with the AAPM TG-218 action limit. The TG-218 action limit at 90% highlights that most plans satisfied institutional acceptance criteria when evaluated using 4%/1 mm and 3%/2 mm, whereas stricter spatial tolerances resulted in reduced compliance under 3%/1 mm.

### Correlation between gamma passing rate and plan modulation

**Fig. 8:**
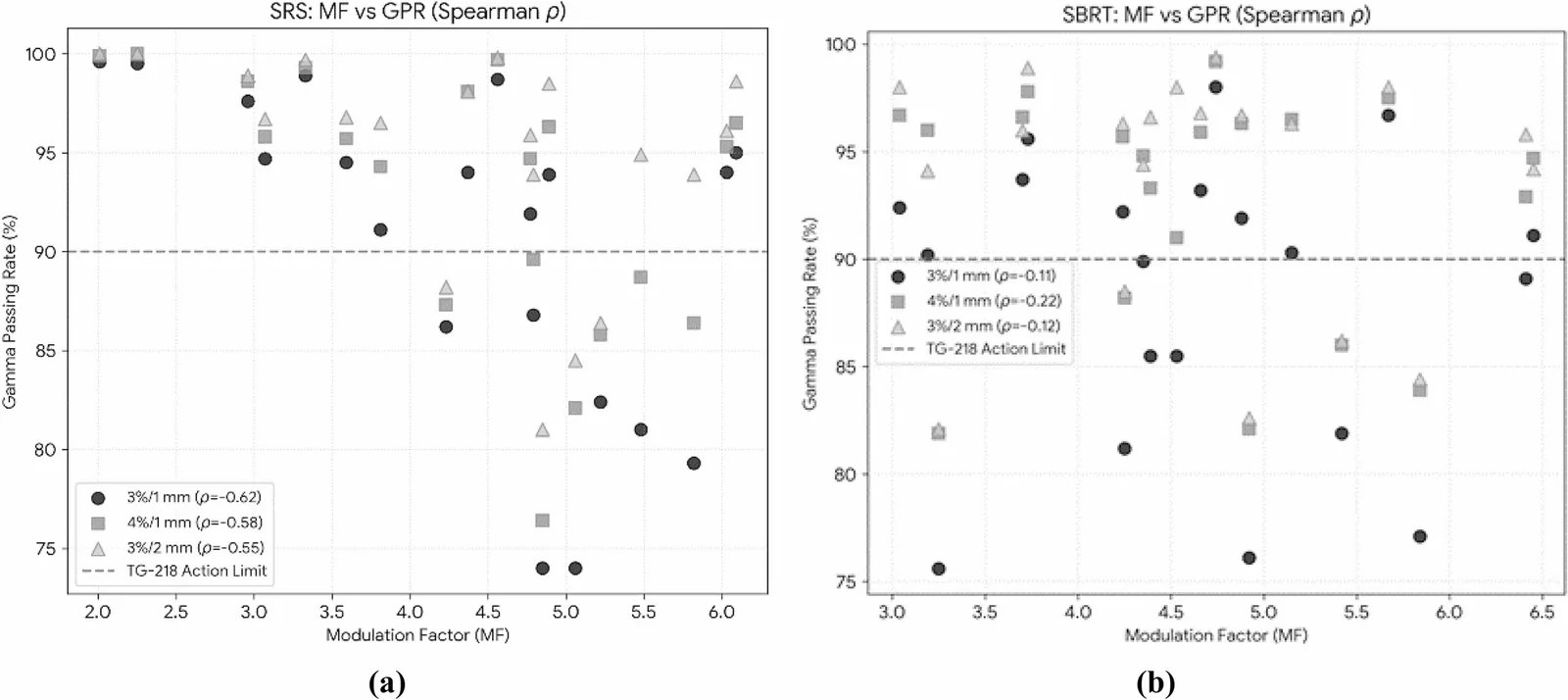
Correlation between MF and GPR for (**a**) SRS and (**b**) SBRT plans. Scatter plots illustrate the relationship across three gamma criteria, with the dashed line indicating the TG-218 90% action limit

The relation between plan modulation and patient-specific QA performance, assessed using the myQA SRS detector, was examined by linking the MF to the GPR by Spearman’s rank correlation analysis (Fig. 8). A strong and statistically significant negative monotonic correlation was detected between MF and GPR for all examined gamma criteria for SRS plans (*n* = 20), showing that enhanced plan modulation correlated with diminished gamma agreement. The most robust connection was identified with the most rigorous criterion, 3%/1 mm (ρ = −0.620, *p* = 0.0035), succeeded by 4%/1 mm (ρ = −0.583, *p* = 0.0069) and 3%/2 mm (ρ = −0.555, *p* = 0.0112). This trend was also apparent at the individual plan level. Highly modulated SRS plans, especially SRS7 (MF = 6.09) and SRS20 (MF = 5.82), demonstrated reduced GPR values under the 3%/1 mm criterion (79.3% in both cases), whereas plans with lower modulation, such as SRS4 (MF = 2.25) and SRS12 (MF = 2.01), achieved GPR values exceeding 99% across all examined criteria.

In contrast, SBRT plans (*n* = 20) exhibited a weak and non-significant correlation between MF and GPR, with correlation coefficients of ρ = −0.112 for 3%/1 mm, ρ = −0.215 for 4%/1 mm, and ρ = −0.117 for 3%/2 mm (*p* > 0.05 for all). The data indicate that, within the modulation range reported in this cohort, SBRT QA performance exhibited less sensitivity to differences in plan complexity relative to SRS. When SBRT plans were stratified by treatment site, spine SBRT cases (*n* = 16) demonstrated the highest degree of modulation, with a mean MF of 4.80 ± 0.97, compared with lung SBRT plans (*n* = 3), which exhibited a lower mean MF of 4.29 ± 0.90. The remaining extracranial SBRT case (mediastinum/adrenal/bone) exhibited a lower MF of 3.19. Despite the higher modulation observed in spine SBRT, the majority of cases achieved GPR ≥ 90%, consistent with the AAPM TG-218 action limit, when evaluated using the 4%/1 mm and 3%/2 mm criteria. In contrast, a larger proportion of spine SBRT plans fell below this threshold under the more stringent 3%/1 mm criterion. The statistical relationship between plan characteristics and dosimetric verification is presented in Table 4.

**Table 4:** Correlation analysis between Modulation Factor (MF) and Gamma Passing Rates (GPR), and Gradient Agreement (R2) results. Note: MF = Modulation Factor; GPR = Gamma Passing Rate. Values in the GPR columns represent Spearman’s rank correlation coefficient (ρ). Gradient Agreement (R2) values represent the mean coefficient of determination from dose profile analysis in high-gradient regions. Asterisks (*) denote statistical significance (*p* < 0.05).*

Plan Group	MF vs. GPR (3%/1 mm)	MF vs. GPR (4%/1 mm)	MF vs. GPR (3%/2 mm)	Gradient Agreement (R2)
SRS (*n* = 20)	-0.620*	-0.583*	-0.555*	0.996
SBRT (*n* = 20)	-0.112	-0.215	-0.117	0.994
All Plans (*n* = 40)	-0.391*	-0.422*	-0.383*	—

An analysis of all SRS and SBRT plans combined (*n* = 40) revealed a moderate and statistically significant inverse correlation between MF and GPR across all gamma criteria (ρ = −0.391 for 3%/1 mm, ρ = −0.422 for 4%/1 mm, and ρ = −0.383 for 3%/2 mm; *p* < 0.05 for all). Across all plan groups, the 3%/1 mm criterion demonstrated the greatest sensitivity to modulation effects, resulting in the highest proportion of plans approaching or falling below the TG-218 action limit, whereas the 4%/1 mm and 3%/2 mm criteria showed improved robustness and higher overall compliance with TG-218 thresholds.

### Dose gradient agreement between TPS and measured dose distributions

**Fig. 9:**
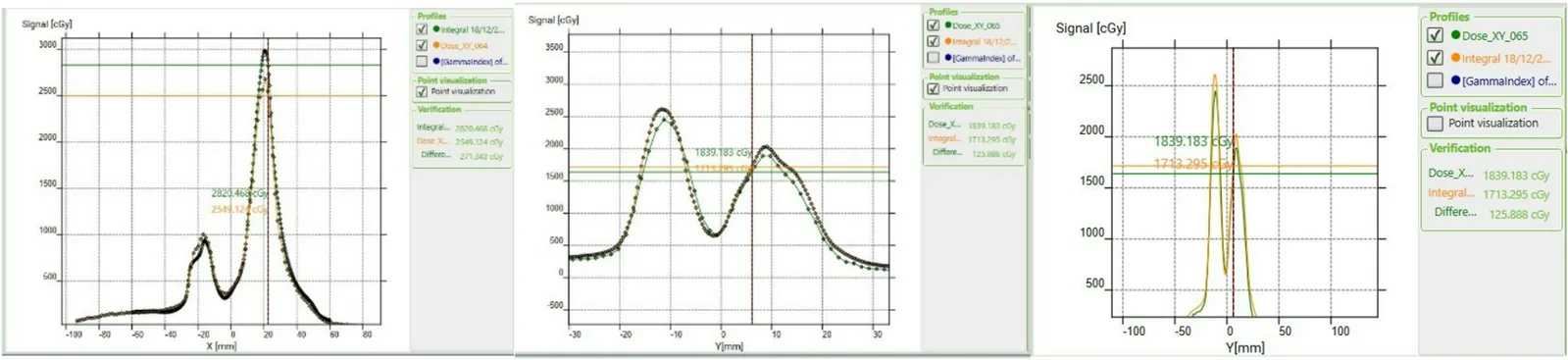
Representative one-dimensional dose profile comparisons between TPS-calculated and myQA SRS-measured dose distributions for selected SRS and spine SBRT plans, illustrating agreement in high-dose regions and steep dose-gradient zones

To assess the ability of the myQA SRS detector to accurately resolve steep dose gradients, one-dimensional dose profile comparisons were performed for five SRS cases involving multiple brain metastases (SRS 1, 4, 7, 12, and 20) and five spine SBRT cases (SBRT 3, 6, 8, 9, and 14). Dose profiles were extracted across clinically relevant high-gradient regions, including dose fall-off between closely spaced intracranial targets and PTV-organ-at-risk (OAR) interfaces, such as the PTV-spinal cord boundary in spine SBRT. R² values ranged from 0.91 to 0.998, with mean values of 0.996 for SRS and 0.994 for SBRT. For the SRS cases including multiple brain metastases treated with single isocenter plans, the detector demonstrated excellent agreement with TPS-calculated dose profiles in regions characterized by complex inter-lesion gradients. Linear regression analysis yielded a mean coefficient of determination (R²) of 0.996, with a mean slope (a) of 0.992 and a mean intercept (b) of 0.42%. High agreement was maintained even for highly modulated plans; for example, SRS 7 (MF = 6.09) achieved an R² of 0.995, while SRS 20 demonstrated a near-perfect R² of 0.998 for metastases separated by approximately 5 mm. Similarly, spine SBRT cases showed strong correspondence between measured and TPS-calculated dose profiles when profiles were oriented along the anterior-posterior axis to evaluate the steep dose gradient at the PTV-spinal cord interface. Across the five SBRT cases, the mean R² was 0.994, with a mean slope of 0.989 and a mean intercept of 0.58%. Both lumbosacral (SBRT 3 and 6) and thoracic (SBRT 8 and 9) plans revealed precise reproduction of the planned steep dose gradient as optimized in the treatment planning system for spinal cord protection, with SBRT 14 reaching a R² value of 0.996. Overall, the consistently high R² values approaching unity, together with slopes close to 1.0 and minimal intercepts, confirm the capability of the myQA SRS detector to accurately measure steep dose gradients in both intracranial and extracranial stereotactic treatments. Figure 9 illustrates that the typical dose profiles exhibit a significant concordance between the dose distributions computed by the TPS and those observed, particularly in high-dose regions and those with steep gradients for three SRS plans. The findings validate that the sub-millimetre detector spacing and CMOS technology significantly reduce partial volume effects and can precisely measure the rapid dose fall-off, which is crucial for protecting organs at risk structures in SRS and SBRT.

## Discussion

The implementation of stereotactic treatments, including SRS and SBRT, requires a paradigm shift in PSQA, moving beyond conventional 3%/3 mm criteria toward more stringent tolerances that reflect the high spatial and dosimetric precision of these techniques. This work thoroughly assessed the efficacy of the myQA SRS detector, a high-resolution CMOS-based array, within a cohort of complex intracranial and extracranial stereotactic plans. Prior to clinical plan evaluation, small-field output consistency was verified to ensure agreement between measured and TPS-calculated dose values. In this context, ionization chamber measurements were used as a consistency check of the TPS beam model rather than for absolute small-field dosimetry, and no additional correction factors were applied. A similar approach has been reported by Junis et al. [15], where small-field measurements were used to confirm agreement with TPS calculations under clinical conditions. This pragmatic strategy supports the reliability of the dosimetric chain before proceeding to patient-specific QA analysis. The reported ranges of included cases reflect the clinical heterogeneity of the study cohort, with SRS plans including predominantly small intracranial lesions alongside occasional larger targets, whereas SBRT plans encompassed substantially larger extracranial target volumes with broader variability. The results demonstrate that while the detector achieves high overall compliance with AAPM TG-218 recommendations, QA sensitivity to plan modulation and steep dose gradients varies substantially depending on treatment site and gamma criteria. A principal finding of this work is the statistically significant inverse relationship between plan modulation and gamma passing rates (GPRs) for SRS plans. For SRS cases (*n* = 20), a moderate negative monotonic correlation was observed between the MF and GPR across all evaluated gamma criteria, with the strongest association occurring for the most stringent 3%/1 mm criterion (ρ = −0.620, *p* = 0.0035). This indicates that increased plan complexity-particularly in single-isocenter multiple-target (SIMT) SRS-corresponds to reduced agreement between measured and TPS-calculated dose distributions. Similar trends have been reported by Zhou et al. and Stepanek et al., who demonstrated that highly modulated plans amplify sensitivity to MLC positioning uncertainties and small-field modeling limitations, particularly when evaluated with high-resolution QA devices [5, 9]. Timáková and Zavgorodni further showed that off-axis and multi-target stereotactic plans exhibit reduced gamma performance with increasing modulation, reinforcing the clinical relevance of MF as a complexity surrogate in SRS QA [17]. In contrast, SBRT plans in this study showed weak and non-significant correlations between MF and GPR across all criteria (ρ ≥ −0.22, *p* > 0.05). This decreased sensitivity to modulation is likely due to increased target volumes and adjacent critical organs, which require strict dose constraints, particularly in single-fraction stereotactic treatmen, reduced MLC modulation, and varying beam geometries often employed in extracranial stereotactic therapies. When stratified by treatment site, spine SBRT had the largest modulation (mean MF = 4.80 ± 0.97); however, the majority of patients adhered to the TG-218 action limit when assessed using 4%/1 mm and 3%/2 mm criteria. Yet the 3%/1 mm criterion provided better sensitivity, identifying a higher proportion of spine plans that approached or dropped below the 90% limitation. These findings corroborate previous research highlighting the necessity for stricter criteria to adequately encompass clinically significant delivery uncertainty in areas proximal to serial organs, such as the spinal cord [17, 19–21].

The choice of gamma criteria plays a critical role in interpreting PSQA results for stereotactic treatments. In this study, the 3%/1 mm criterion consistently demonstrated the greatest sensitivity to modulation effects and steep dose gradients, whereas 4%/1 mm and 3%/2 mm criteria showed improved robustness and higher overall passing rates. This outcome aligns with prior studies comparing high-resolution detector arrays to radiochromic film, which demonstrated that stricter spatial tolerances more accurately represent actual delivery precision without artificially increasing passing rates [12, 17]. The sub-millimeter detector spacing (0.4 mm) of the myQA SRS array substantially reduces volume-averaging effects, a known drawback of broader diode arrays as highlighted by Ahmed et al. [20]. The PSQA measurements for both SRS and SBRT utilizing the myQA SRS detector indicated comparable spatial patterns of gamma agreement, with elevated gamma values primarily located in regions with significant dose gradients. Localized inconsistencies were primarily noted at the PTV-OAR interfaces or between neighbouring targets, whereas areas of high-dose plateau consistently demonstrated improved concordance. Significantly, gamma performance was enhanced under less rigorous standards, suggesting that the identified faults were primarily gradient-induced rather than due to systematic dosage inaccuracies. This spatial pattern underscores the therapeutic imperative of combining high-resolution detector outcomes with rigorous gamma criteria to precisely evaluate stereotactic dose administration. The detector’s ability to identify rapid dose gradients was further confirmed using one-dimensional dose profile analysis. These results validate the precise replication of rapid dose decline areas essential for safeguarding nearby organs at risk. Similar findings were reported by Berk et al., who demonstrated that high-resolution arrays are capable of capturing steep gradients with accuracy comparable to radiochromic film [21]. This study proposes the implementation of site-specific and detector-appropriate action limits instead of universal thresholds, based on these findings. Although TG-218 offers a comprehensive framework for IMRT and VMAT quality assurance, its guidelines do not specifically consider the increased sensitivity of stereotactic treatments assessed with sub-millimeter detectors. Proposed proactive clinical action limits, based on observed distributions, are around 94% for SRS and 93% for SBRT using the 3%/1 mm criterion, aimed at identifying modest delivery mistakes while minimizing false failures. A 3%/2 mm criterion of 97% serves as a reliable standard for total delivery accuracy. The observed inverse relationship between plan complexity and gamma passing rates in intracranial SRS plans aligns with the model recently proposed by Hernandez et al. [22]. Their research posits the MF as a definitive and easily understood metric for aperture modulation. This work further illustrates that plans characterized by high complexity are notably less robust when subjected to errors in machine delivery and the constraints of the treatment planning system’s modeling. This statistical correlation underscores the claim that clinical “plan quality” must include both dosimetric performance and delivery robustness. To address the limitations of universal thresholds, we propose site-specific action limits of 94% for SRS and 93% for SBRT at the 3%/1 mm criterion. The suggested site-specific action limits (94% for SRS and 93% for SBRT at the 3%/1 mm criterion) were determined using the observed gamma passing rate distribution and institutional performance, reflecting attainable yet clinically relevant thresholds for high-resolution stereotactic quality assurance. To ensure reproducibility for other clinics, these thresholds were derived using a distribution-based approach, specifically calculated from the mean gamma passing rate minus one standard deviation of the observed results for each cohort. Integrating metrics such as MF and GI into the clinical workflow allows for a proactive quality assurance paradigm that accounts for the unique precision requirements of high-gradient stereotactic delivery. This demonstrates that the universal 90% threshold advised by TG-218, which might not be adequate sensitivity for recently developed radiosurgery dedicated high-resolution detectors, is augmented with rigorous site-specific tolerances that reflect a baseline of institutional process competence.

Recent advances in EPID-based PSQA have also been proposed as an alternative approach for stereotactic QA. Hajare et al. demonstrated that high-resolution EPID-based 2D and 3D automated QA systems can be clinically implemented for SRS and SBRT when rigorously commissioned and supported by advanced dose reconstruction algorithms [23]. However, guidance from AAPM Task Group 307 emphasises that EPID dosimetry requires careful modeling of detector response, beam energy dependence, and backscatter effects, and that its sensitivity in very small fields and steep dose-gradient regions may be limited without extensive commissioning [24]. As a result, while EPID-based QA can offer workflow efficiency and automation benefits in well-equipped clinics, high-resolution detector arrays and film dosimetry remain particularly valuable for stereotactic applications where sub-millimeter spatial accuracy and reliable gradient verification are critical.

The study’s limitations include the limited sample size for film cross-validation (*n* = 9), which was used as an institutional benchmark due to the practical constraints of film dosimetry. Although the dataset size prevents formal significance testing for this particular comparison, high spatial concordance and perfect ranking correlation were observed. In addition, the relationship between plan complexity and high-resolution stereotactic verification should be studied in more detail through multi-institutional research. In future we aim to extend this research with including a variety of energies and delivery systems, since this study only looked at 6 MV FFF beams on one platform.

Finally, this work reinforces the role of the myQA SRS detector as a practical alternative to radiochromic film for routine stereotactic PSQA. Although film remains the gold standard for spatial resolution, its labour-intensive workflow limits its clinical practicality. The myQA SRS system’s real-time readout, extensive active area, and elevated spatial resolution yield significant efficiency improvements, especially for SIMT SRS schemes requiring the capture of many lesions in a single measurement [12, 17, 19]. The findings indicate that the myQA SRS, when used with sufficiently rigorous criteria, provides a dependable and highly sensitive framework for validating contemporary stereotactic radiation delivery.

## Conclusion

This study confirms that the high-resolution CMOS detector is a strong and efficient alternative to film dosimetry for stereotactic PSQA. The sub-millimeter spatial resolution is essential for accurately resolving the steep dose gradients and small-field penumbras characteristic of contemporary SRS and SBRT delivery. Our investigation substantiates that plan complexity significantly influences delivery accuracy, particularly in intracranial SRS, highlighting the necessity for high-resolution arrays to identify subtle delivery errors in sophisticated, highly modulated plans. Because universal tolerance limits recommended by TG-218 may be insufficiently sensitive for high-precision stereotactic verification, we advocate for the adoption of more rigorous, site-specific action thresholds. By adhering to these standards, the verification system’s accuracy aligns with the technical demands of stereotactic delivery, thereby establishing a proactive structure that harmonizes dosimetric validation with the efficiency of clinical processes.

## Supplementary Information

Below is the link to the electronic supplementary material.

Supplementary Material 1.

Supplementary Material 2.

Supplementary Material 3.

## Data Availability

The data will be made available upon request. The necessary information can be obtained from the corresponding author.
