# Supplementary material for: Clinical assessment of dedicated SRS detector for dose verification in SBRT/SRS treatment plans: to establish clinical threshold with AAPM TG-218 recommendation
Source: Ir J Med Sci. 2026 Apr 14;195(4):1853–67. doi: 10.1007/s11845-026-04379-y (PMC13588844; doi:10.1007/s11845-026-04379-y)
Supplement: Supplementary file 3 [file 11845_2026_4379_MOESM2_ESM.docx]

**Conflict of Interest Statement:**

To whom it may concern,

### We have not had any actual or potential conflict of interest for the article titled “Clinical Assessment Of Dedicated SRS Detector For Dose Verification in SBRT/SRS Treatment Plans: To Establish Clinical Threshold with AAPM TG-218 Recommendation” that we have submitted to you for publishing. We confirm that the material has not been published previously, and will not be submitted for publication elsewhere.

Regards,

Cemile Ceylan declares that she has no conflict of interest for his research.

Osman Artunç Türe declares that he has no conflict of interest for his research.
